# Hepatitis A and hepatitis B infection risk among employees at schools for disabled pupils

**DOI:** 10.1038/s41598-022-24579-7

**Published:** 2022-11-23

**Authors:** Felix Lang, Klaus Schoene, Felix Goessler, Dirk-Matthias Rose, Peter Kegel

**Affiliations:** 1 Institute of Occupational, Social and Environmental Medicine, University Medical Center of the Johannes Gutenberg University Mainz, Obere Zahlbacher Straße 67, 55131 Mainz, Germany; 2 Institute of Teacher’s Health, University Medical Center of the Johannes Gutenberg University Mainz, Kupferbergterrasse 17-19, 55116 Mainz, Germany

**Keywords:** Hepatitis B virus, Occupational health

## Abstract

Aim of this project was to assess occupational biological hazards with regard to the risk of hepatitis A (HAV) and hepatitis B (HBV) and the need for occupational health care in schools for pupils with special needs. Teachers and educational specialists were surveyed about activities potentially providing exposure to biological agents and their individual immune status regarding HAV and HBV by a detailed questionnaire. Descriptive analyses, group comparisons and logistic regression were performed to identify factors influencing the HAV and HBV immune status. 1398 teachers and educational specialists took part. 1381 respondents reported having physical contact with pupils at work (98%). Daily contact was reported by up to 84% of all employees. Being scratched, bitten or spat at was reported by up to 93%. Hazardous activities are performed by both teachers and educational specialists. The vaccination rate was reported to be 58% for HAV and 64% for HBV. In regression analyses, failing to receive vaccine counselling [HAV: aOR 0.36 (95% CI 0.28; 0.46; p < 0.001), HBV: aOR 0.43 (95% CI 0.33; 0.55; p < 0.001)] or non-participation in infection prevention instruction [HBV: aOR 0.54 (95% CI 0.39; 0.75; p < 0.001)] were found to be significant predictors of low vaccination rates. Employees who are at risk due to occupational exposure should be instructed about infection prevention and vaccination against HAV and HBV.

## Introduction

Viral hepatitis is one of the most common infectious diseases worldwide. Death rates are comparable to deaths caused by tuberculosis or HIV and continue to increase over time. Available and effective vaccines can prevent hepatitis A (HAV) and hepatitis B (HBV) infections^1^. In advance, various studies have described an increased prevalence of HBV among pupils and teachers in special schools for handicapped children and an increased seroconversion rate compared to the general population^2–4^.

In order to address the individual support needs of pupils with disabilities, there are special schools in Rhineland-Palatinate, Germany, with different special focuses. Support focuses can be, for example, mental and holistic development, physical development, social-emotional development, learning disabilities as well as combinations of these focuses and interdisciplinary support centres. Claus et al.^5^ previously came up with the results of a cross-sectional study at 13 special schools with a focus on physical and/or holistic development. Particularly at special schools with a focus on the combination of mental and physical development, where some severely and multiply disabled pupils are often in close physical contact with the supervising educational specialists and teachers, there are particularly favourable conditions for the transmission of pathogens. However, there was a notion of an increased need for prevention also at schools with a special focus on social-emotional development with interdisciplinary focus. Aggressive behaviour, e.g. scratching, biting and spitting by pupils, had previously been reported and remains a risk for infectious exposure.

In order to minimise the work-related risk of infection for employees in schools, the Institute for Teacher’s Health carried out a risk assessment at different types of special schools in Rhineland-Palatinate, especially with regard to the risk of infection by the HAV and HBV.

## Methods

The present study was conducted as a cross-sectional online survey held between March to July 2016. 1.535 active teachers and educational specialists of 38 state special schools in Rhineland-Palatinate were asked to participate in the risk assessment with the focus on infectious diseases and infection prevention. The schools were categorised according to different support priorities. A distinction is made between schools with a special focus on physical (further named P-schools), mental (M-schools) or social-emotional development (SE-schools) and support centres with an interdisciplinary focus (IF-schools). The pupils at these schools are usually between 6 and 18 years old.

The risk assessment questionnaire developed by the Institute of Teachers' Health at the University Medical Center of the Johannes Gutenberg University Mainz included the following topics:

- “Occupation-related risk of infection”: How often are nursing activities such as intimate care or first-aid measures carried out, and how often do hazardous situations such as scratching, biting or spitting by schoolchildren occur?
- "Infectious diseases": Do immunity to HAV and HBV exist due to vaccination or to previous illness (by self-disclosure of the respondents)?
- "Information-based infection protection": This includes vaccination counselling within the last five years by physicians, health insurance companies or the public health department. These may have been carried out as part of occupational health care or in another context. It was also recorded whether infection protection instruction is offered at the school every two years and whether the employee participated in the last infection protection instruction.

With regard to vaccination status, the participants were divided into three age groups. The age cut-off at 45 years corresponds to the average age of the participants. For pragmatic reasons, an additional age limit was chosen, as a higher vaccination rate was to be expected in the group of under 30-year-olds according to the WHO recommendation of 1992 to vaccinate children against HBV. Before performing the statistical analyses, the different schools were divided into two groups according to their particular special focus of support: like in the study by Claus et al., M-schools and P-schools were considered together, as were SE-schools and IF-schools, since biological hazards do not appear obvious here. Descriptive statistics and significance tests (Chi^2^ test, two-sided, if necessary Fisher-Yates-Test) were used to describe the sample and to record group differences. It was tested to what extent the data from teachers vs. educational specialists or the data from staff in M- and P-schools versus the data from staff in SE- and IF-schools systematically differ. Multivariate logistic regression analyses were used to determine possible influencing factors of the HAV and HBV-related vaccination status. Cases of persons already suffering from HAV or HBV were not considered.

Dichotomous or dichotomised variables were used to conduct the statistical evaluations (never vs. ever). For this purpose, "never" entries were coded with “0” for the variables on the work-related risk of infection, all other frequency entries ("very rarely/ 1–2 times per school year" to "always/multiple times a day") were coded with “1”. For the variables “vaccination counselling” and “infection prevention instruction”, the answers "yes" and "rather yes" were coded with “1″, the answers "no" and "rather no" and missing information with “0″. Immunities due to vaccination or previous illnesses were recorded separately (applicable = “1″, other/no information = “0″). The significance level was determined with p = 0.05.

For better readability, all percentages have been rounded to full integers where applicable. Statistical analyses were performed using IBM SPSS Statistics 23.0.0.3.

### Ethics approval and consent to participate

The present data were collected and analyzed anonymously by the Institute of Teacher’s Health (IfL) in the course of fulfilling its legal mandate to carry out occupational health care for all employees in the state school service in Rhineland-Palatinate, Germany. The Ethics Committee of the Rhineland-Palatinate State Medical Association, Germany, has given its approval and confirmed that no further measures are required for the evaluation of anonymized data collected as part of the fulfillment of the IfL's statutory work mandate The data protection officer's approval has been obtained.

## Results

**Figure 1:**
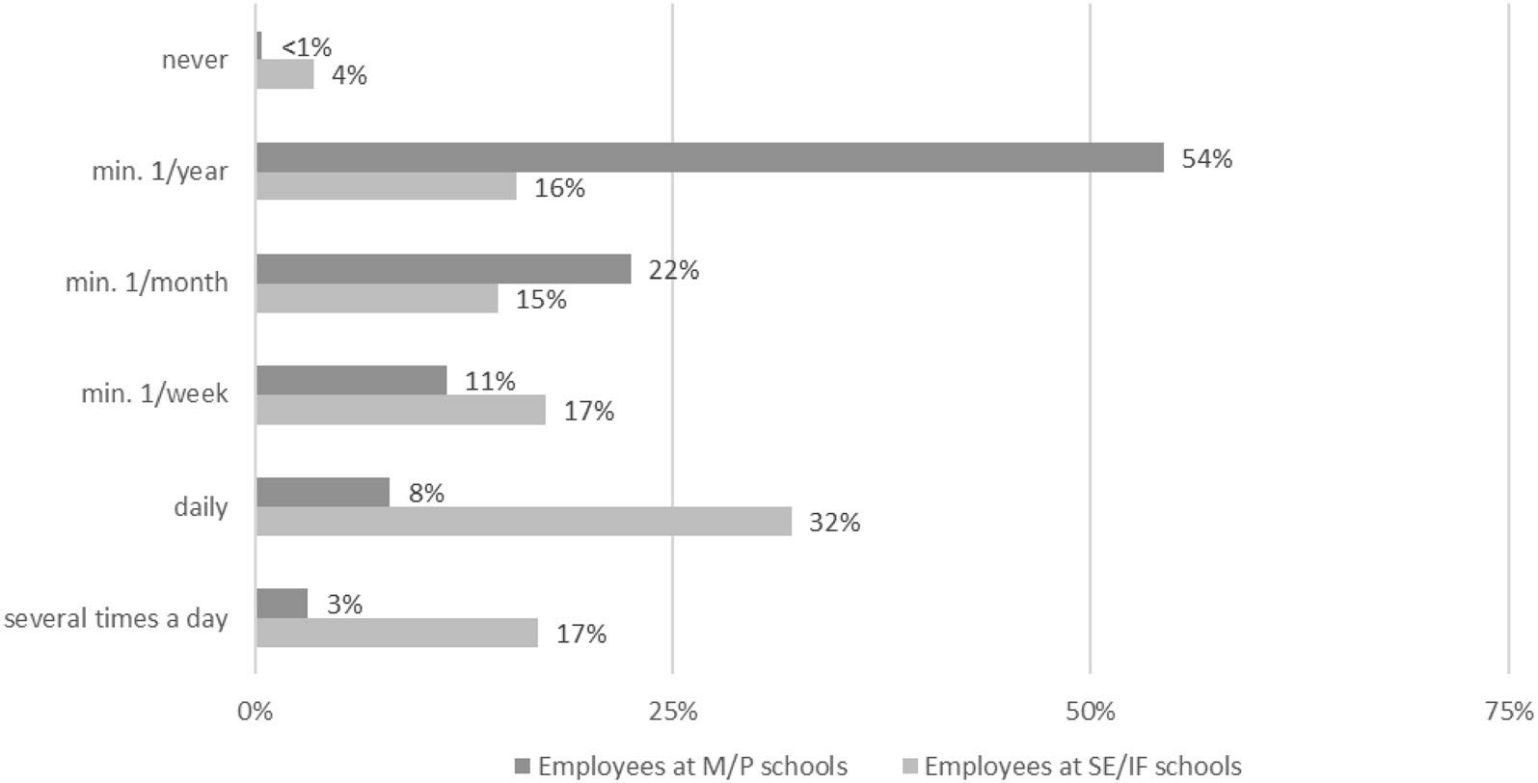
Nursing activities according to the schools’ support focus. Frequency of all types of nursing performed according to the focus of the schools. Percentages are based on the total number of employees, teachers and educational specialists, in the respective group (M/P schools: n = 943; SE/IF schools: n = 455).

91% of the invited employees took part in the survey (1.398/1.535), of which 39% were teachers (TS) and 61% educational specialists (ES). 83% were female; the mean age was 45.8 years (SD 10.9). 943 participants (68%) were employees in M and P schools, 455 (33%) were employees in SE and IF schools. Almost all respondents, i.e. 96% of the teachers at SE and IF schools and 100% of the educational specialists at M and P schools, stated that close physical contact with pupils generally occurs. 81% of the teachers and 86% of the educational specialists at M- and P-schools stated that this occurs daily. At SE- and IF-schools daily contact only applies to 9% of the teachers and 38% of the educational specialists respectively. Beyond just physical contact when providing general assistance, Fig. 1 shows how frequently nursing activities such as treating injuries, intimate hygiene, or assisting with medication are performed at the surveyed schools. Only 4% of employees at SE/IF schools reported not performing any nursing activities. Nearly 100% of employees at M/P schools perform such activities at least once a year or more often (Fig. 1).

A total of 48% of the teachers compared to 86% of the educational specialists at SE-schools perform supporting activities when the pupils go to the toilet. Diaper changes or catheterization also occur in all school forms, but with a lower frequency than assistance with toileting. First-aid activities including care of injuries, bleeding wounds, etc. are carried out by 94–98% of the staff, mostly "rather rarely" (once or twice a month) or "less frequently" (annually) (Table 1).

**Table 1:** Frequency of physical contact and hazardous activities performed by school type and occupational group.

Activity	Sup. focus	Prof	Always (several times a day)	Very often (daily)	Rather often (1—2 times/week)	Rather rarely (1–2 times/month)	Very rare (1–2 times/school year)	Applies (always–very rarely)	Not applicable (never)	TS vs. ES	M/P vs. SE/IF
			in %	in %	in %	in %	in %	in %	in %	p*	p*
Close physical contact with pupils (helping, comforting, blowing nose)	M/P	TS	60	21	10	6	3	99	1	0.006/ 0.023	^TS^0.060 ^**ES**^**0**.**000**
		ES	62	24	11	3	1	100	0		
	SE/IF	TS	16	29	25	16	11	96	4	0.440	
		ES	41	29	14	8	5	98	2		
Treatment of injuries (e.g. bleeding wounds) in pupils	M/P	TS	< 1	2	16	47	33	98	2	0.735	^**TS**^**0**.**030** ^ES^0.962
		ES	1	3	21	51	23	98	2		
	SE/IF	TS	0	1	12	47	35	95	5	0.111	
		ES	0	6	21	44	27	98	2		
Supporting pupils in visiting the toilets/ intimate hygiene	M/P	TS	36	21	15	12	8	93	7	0.001	^TS^0.000 ^ES^0.000
		ES	47	17	14	13	6	98	2		
	SE/IF	TS	4	5	6	12	21	48	53	0.000	
		ES	24	14	11	19	17	86	14		
Changing diapers of pupils	M/P	TS	31	19	14	13	13	89	11	0.009	^TS^0.000 ^ES^0.000
		ES	43	15	12	14	9	94	6		
	SE/IF	TS	3	4	5	5	14	32	68	0.000	
		ES	23	9	7	12	17	69	31		
Catheterisation of pupils	M/P	TS	0	0	0	< 1	5	5	95	0.007	^**TS**^**0**.**003** ^ES^0.100
		ES	4	1	1	1	3	10	90		
	SE/IF	TS	0	0	0	< 1	< 1	1	99	0.001	
		ES	0	0	< 1	2	3	6	94		
Probing pupils	M/P	TS	8	4	4	5	8	29	71	0.560	^TS^0.000 ^ES^0.000
		ES	10	5	3	7	7	31	69		
	SE/IF	TS	< 1	< 1	< 1	1	1	4	96	0.000	
		ES	5	2	1	2	6	15	85		
Supporting pupils in the intake of food	M/P	TS	29	22	14	12	12	90	11	0.196	^TS^0.000 ^ES^0.000
		ES	38	22	12	11	9	92	8		
	SE/IF	TS	3	3	5	8	15	34	66	0.000	
		ES	20	13	8	11	17	69	31		
Supporting pupils in taking medication	M/P	TS	15	20	14	16	17	82	18	0.225	^TS^0.000 ^ES^0.000
		ES	21	23	9	13	20	85	15		
	SE/IF	TS	2	6	6	8	25	47	53	0.000	
		ES	14	11	9	11	23	69	31		
Being scratched, bitten or spat at by pupils	M/P	TS	4	8	19	32	28	90	10	0.074	^TS^0.000 ^ES^0.000
		ES	8	12	18	27	30	94	6		
	SE/IF	TS	0	1	5	10	43	60	40	0.000	
		ES	2	7	13	23	32	77	23		

Special schools with focus on mental and holistic development (M) and physical development (P): teachers (TS): N = 267, educational specialists (ES): N = 674; special schools with focus on social-emotional development (SE) and interdisciplinary focus (IF): TS: N = 280, ES: N = 175.

*Significance test (Chi^2^ test).

Between TS and ES, particularly at SE/IF special schools, there are significant differences in terms of activity, e.g. in the support of pupils for toilet visits and intimate hygiene (P/M: 93% (TS) vs. 98% (ES); SE/IF: 48% (TS) vs. 86% (ES)) as well as in changing pupils' diapers (P/M: 89% (TS) vs. 94% (ES); SE/IF: 32% (TS) vs. 67% (ES)). Catheterization, but also probing, of pupils by staff is generally rare: TS at SE/IF special schools are the least affected (1% and 4% respectively). Being scratched, bitten or spat on by pupils was reported by 90% of the TS and 94% of the ES at M/P special schools and 60% of the TS and 77% of the ES at SE/IF special schools. This is described by the majority as occurring "rather rarely" or "very rarely". Pupils at SE-/IF special schools need to be supported in taking medication significantly less often than at M-/P- special schools. The majority of drugs are administered by ES (Table 1).

78% of the TS and 71% of the ES at M-/P- schools as well as 60% of the TS and 60% of the ES at SE-/IF- schools participated in a vaccination counselling within the last 5 years. The participation rates for infection prevention instruction within the last two years are significantly lower with 54% of the TS and 50% of the ES at M-/P- schools and 24% of the TS and 33% (ES) at SE-/IF- schools. 50% of the TS and 44% of the ES at M-/P-Special Schools and 29% of the TS and 27% of the ES at SE-/IF- Special Schools stated that infection protection instruction was offered at the school every two years (Table 2).

**Table 2:** Participation in vaccination counselling and Infection protection advice.

Counselling/instruction	Support focus	Profession	(Rather) yes (%)	(Rather) no/unstated (%)	TS vs. ES (p*)	M/P vs. SE/IF (p*)
Vaccination counselling within the last 5 years	M/P	TS	78	22	0.033	^TS^0.000 ^ES^0.005
		ES	71	29		
	SE/ IF	TS	60	40	0.940	
		ES	60	40		
Infection prevention instruction at school every 2 years	M/P	TS	50	50	0.130	^TS^0.000 ^ES^0.000
		ES	44	56		
	SE/ IF	TS	29	71	0.692	
		ES	27	73		
Participation in the last infection protection instruction	M/P	TS	54	46	0.189	^TS^0.000 ^ES^0.000
		ES	50	50		
	SE/ IF	TS	24	76	0.032	
		ES	33	67		

Comments: special schools with focus on mental and holistic development (M) and physical development (P): teachers (TS): N = 267, educational specialists (ES): N = 674; special schools with focus on social-emotional development (SE) and interdisciplinary focus (IF): TS: N = 280, ES: N = 175.

*Significance test (Chi^2^ test).

A total of 2% and 1% of the participants stated that they had been infected with HAV or HBV before. ES are more frequently affected by HAV infections (2% vs. 1%) and HBV infections (2% vs. 1%, n. sign.) than TS. (Table 3).

**Table 3:** HAV and HBV vaccination and immune status.

		N	HAV vaccination	HBV vaccination	Undergone HAV (p*)	Undergone HBV (p*)
Age	20–29 J	123	72%^a^ (p = 0.001)	81%^a^ (p = 0.000)	0% (p = 0.142; p_Fi_ = 0.251)	1% (p = 0.717; _pFi_ = 1.000)
	30–44 J	525	62%	67%	0%	< 1%
	≥ 45 J	750	53%^b^ (p = 0.000)	60% (p = 0.000)	3% (p = 0.000; p_FI_ = 0.000)	2% (p = 0.026; p_FI_ = 0.040)
Gender	Female	1161	59% (p = 0.046)	66% (p = 0.004)	2% (p = 0.467; p_Fi_ = 0.403)	1% (p = 0.633; p_Fi_ = 1.000)
	Male	237	52%	56%	2%	1%
Profession^1^	ES	849	60% (p = 0.062)	67% (p = 0.014)	2% (p = 0.013)	2% (p = 0.092)
	TS	547	55%	60%*	1%	1%
Support focus	M/P	943	65% (p = 0.000)	71% (p = 0.000)	2% (p = 0.941)	1% (p = 0.517)
	ES/IF	455	44%	50%	2%	1%

Comments: self-report of respondents.

*M* special schools with focus on mental and holistic development, *P* special schools with focus on physical development (P), *IF* special schools with focus on social-emotional development, *IF* special schools with interdisciplinary focus, *TS* teachers, *ES* educational specialists.

*Significance test (Chi^2^-Test, Fisher-Yates-Test: pFI).

^a^20–29-year-old vs. older (30 +).

^b^ ≥ 45-year-old vs. younger (U45).

^1^2 persons without information on occupational group.

According to the self-reports of the respondents, the average HAV vaccination rate is 58%, the HBV vaccination rate 64%. Those over 45 years of age show significantly lower vaccination rates than those under 45 years of age. The highest vaccination rate was found among participants younger than 30 years. The age-related odds ratios from the multivariate model are aOR 0.98 (0.96; 0.99) for HAV and aOR 0.97 (0.96; 0.98) for HBV. Women reported being vaccinated against HAV and HBV significantly more frequently than men (HAV: 59% vs. 52%; HBV: 66% vs. 56%). However, in the multivariate model, a significantly lower probability for men of being vaccinated could only be demonstrated for HBV: aOR 0.63 (0.46; 0.87) (Tables 3, 4).

**Table 4:** Results of logistic regression analyses.

	HAV vaccination^a^ N = 1.374	HBV vaccination^b^ N = 1.380
	aOR (95% CI)	aOR (95% CI)
Age	0.98 (0.964; 0.986)	0.97(0.962; 0.985)
SE/IF vs. reference group M/P	0.55 (0.411; 0.748)	0.58 (0.425; 0.785)
Professional group TS vs. reference group ES	0.96 (0.737; 1.250)	1.0 (0.76; 1.324)
Men vs. reference group women	0.77 (0.566; 1.054)	0.63 (0.461; 0.867)
No participation in vaccination counselling vs. reference group Participation in vaccination counselling	0.36 (0.276; 0.458)	0.43 (0.331; 0.551)
No offer of infection prevention instruction every 2 years vs. reference group 2-year offer of infection prevention instruction	0.83 (0.613; 1.136)	0.81 (0.582; 1.123)
No Participation in infection prevention training vs. reference group	0.74 (0.539; 1.002)	0.54 (0.389; 0.750)
Support for toilet visits/intimate care vs. reference group	0.69 (0.413; 1.151)	0.57 (0.344; 0.957)
Diapers of pupils vs. reference group	1.40 (0.885; 2.219)	0.93 (0.582; 1.476)
Support with food intake vs. reference group	0.80 (0.523; 1.214)	0.98 (0.633; 1.509)
Support with medication vs. reference group	0.92 (0.671; 1.255)	0.80 (0.581; 1.103)
Catheterisation of pupils vs. reference group	0.94 (0.561; 1.586)	.82 (0.471; 1.445)
Probing of pupils vs. reference group	0.91 (0.662; 1.256)	1.08 (0.769; 1.509)
Being scratched. bitten or spat at by pupils vs. reference group	0.86 (0.594; 1.252)	1.18 (0.800; 1.735)
Nagelkerke R^2^	0.18	0.20

Only variables for which statistically significant correlations were found in bivariate correlation analyses were included.

*M* special schools with focus on mental and holistic development, *P* special schools with focus on physical development (P), *IF* special schools with focus on social-emotional development, *IF* special schools with interdisciplinary focus, *TS* teachers, *ES* educational specialists.

^a^No persons suffering from HAV were included in the analysis.

^b^No persons suffering from BV were included in the analysis.

ES reported being vaccinated against HB significantly more often than TS (67% vs. 60%), but in the multivariate model the occupational group does not represent a statistically significant influencing factor. The vaccination rates reported for both HAV and HBV are significantly lower at SE/IF special schools than at M/P special schools (HAV: 44% vs. 65%; HBV: 50% vs. 71%). The multivariate model confirms this correlation with an aOR of 0.55 (0.41; 0.75) for HAV and an aOR of 0.58 (0.43; 0.79) for HBV. Staff members who do not assist pupils with toilet visits or intimate hygiene are also significantly less likely to be vaccinated against HBV: aOR 0.57 (0.34; 0.96). This was not apparent with regard to HAV: aOR 0.69 (0.41; 1.15).

In addition, staff members who have not participated in a vaccination counselling in the last 5 years are significantly less likely to have been vaccinated compared to staff members who have had previous vaccination counselling [HAV: aOR 0.36 (95% CI 0.28; 0.46; p < 0.001), HBV: aOR 0.43 (95% CI 0.33; 0.55; p < 0.001)]. The same applies to staff members who did not participate in infection prevention instruction (HBV: aOR 0.54 (95% CI 0.39; 0.75; p < 0.001), a similar trend was found for HAV, although not being significant).

## Discussion

### HBV hazards in the workplace

Various studies have previously investigated the prevalence of HBV infections or the seroprevalence of HBV in institutions for the disabled. Most studies found a higher proportion of post-infection conditions (anti-HBc positive) in mentally impaired residents compared to the general population^6–10^. Increased HBV seroconversion rates among teachers have also been described previously, assuming the presence of HBV carriers among pupils^2,3^.

In 2015, the WHO estimated that 4% of the population worldwide live with chronic HBV infection^11^. According to Poethko-Müller et al.^12^, the serostatus in a Germany-wide sample showed HBV infection in approximately 5% of adults. Similar studies from other countries presented comparable information^13,14^. In the present study, 1% and 2% of the respondents stated that they had had HBV and HAV respectively. This discrepancy could be due to the limitation of this study, namely that only self-reports are available but no serological evidence is. In addition, HBV may have a subclinical course.

In the present study, 95–98% of all respondents stated that they were treating injuries among pupils, the majority (71–82%) 1–2 times a month or less. These findings support the recommendation of the German Standing Committee on Vaccination (STIKO) that company first aiders, who are classified by the Committee on Biological Agents (ABAS) of the German Federal Ministry of Labour and Social Affairs (BMAS) as an occupational group with an increased risk of infection, be vaccinated against HBV^15^. However, with regard to first aid activities, there is currently a lack of data to show that there is an increase in the number of accidents in special schools compared with the general population.

Both, teachers and educational specialists, support their pupils in taking medication. This also includes the administration of injections. However, it has not been clarified whether these invasive activities are associated with an increased risk of needlestick injuries. Assuming increased incompliance and decreased impulse control among pupils in care, aggressive behavior such as scratching, biting, or spitting by pupils is not negligible as a potential risk of infection from injury and is reported from all special schools, regardless of the special focus of support. Remis et al. previously described transmissions of HBV from mentally retarded pupils to their teachers. The risk of infection for teachers who report student contact in the classroom is found to be increased more than fourfold^16^. Accordingly, the results of this study indicate that employees at special schools are at increased risk of HBV infection and that HBV prophylaxis is likely to be advised.

### HAV hazards in the workplace

Pupils with mental or physical disabilities are diapered, washed, fed, probed and catheterized by the staff. The regular performance of these activities entails a risk of infection for pathogens that are excreted via the stool. Possible contact can occur here, for example, during assisted toilet use, incontinence care or intimate hygiene. Nevertheless, contact can also occur with nasal secretions, saliva or infected blood during wound care. Claus et al. showed that many pupils at special schools with focus on mental or physical disabilities could not follow elementary hygiene rules, so that body excretions remain on hands, body and objects. There would therefore be an uncontrollable, increased risk of both contact and smear infections and those transmitted via droplet infection^5^.

According to the results of the present study, almost all employees working at special schools with a focus on mental or physical disabilities assist pupils with toilet visits and intimate hygiene at least once a year. Almost every second employee take part in this activity on a daily basis. At special schools with a focus on social-emotional disabilities and with interdisciplinary focus, this applies to significantly fewer workers overall and predominantly to the educational specialists. The frequency of diaper changes is generally lower. The relative distribution of these activities between the occupational groups is comparable at all schools, irrespective of the support focus. However, activities with risk of infection such as catheterization, which one would rather expect in mentally or physically impaired pupils, are also carried out in schools with a focus on social-emotional or interdisciplinary focus, although to a much lesser extent.

In the context of the present survey, about 10% of the employees at special schools with a focus on mental or physical disabilities perform catheterization, predominantly by educational specialists. In contrast, 26% of the respondents in the study of Claus et al.^5^ stated that they catheterized pupils. This difference could be caused by the fact that the study by Claus et al. was based on a self-selective sample of special schools, resulting in the participation of schools mainly for pupils with severe disabilities.

Overall, the activities mentioned here involve contact with potentially infectious faeces or bodily fluids and form part of the activities of all employees of all special schools, regardless of their specialisation. This makes preventive occupational health care including infection prevention counselling and vaccination against HAV highly relevant.

### Vaccination rate

Based on the self-declarations, vaccination rates of 58% (HAV) and 64% (HBV) could be determined in this study. Claus et al.^4^ also reported similar vaccination rates of 42% (HAV) and 80% (HBV) based on vaccination passports and serostatus, which appear to be higher overall than those reported by Poethko-Müller and Schmitz^12^ for the population-wide sample. The finding of the highest HBV vaccination rate in the group of 20–29-year-old persons in all studies is probably a consequence of the vaccination recommendation for infants and children recommended in 1992 by the WHO and issued in Germany in 1995. Moreover, the differences in vaccination rates may be caused by socio-economic effects. For example, Poethko-Müller and Schmitz^12^ found higher vaccination rates among persons with a higher social status.

In the present study, logistic regression analyses were used to determine age, school’s focus of support and participation in vaccination counselling within the past 5 years as significant predictors of HAV and HBV vaccination. For the HBV vaccination, gender, participation in infection protection advice, support of pupils in toilet visits and intimate hygiene were determined as additional significant predictors. Similarly, Claus et al.^4^ found a significantly higher vaccination rate (82% vs. 72%) for HBV for respondents who change diapers compared to respondents who do not change. Staff seem to associate taking on nursing tasks such as changing nappies or assisting schoolchildren with toileting or intimate hygiene with the risk of HBV infection, even though the tasks themselves are mainly associated with the risk of HAV infection. At this point, it is not clear whether the decision to be vaccinated is influenced by subjective risk perceptions or whether this is due to structural guidelines or processes for risk assessment in schools based on corresponding activity or job-related selection mechanisms.

Analogous to the relevant influencing factors identified in the present study, Claus et al.^4^ also pointed out that HAV vaccination is more likely if the employees have been informed about infectious diseases and vaccination protection before they start work. According to the present study, explicit vaccination counselling is more likely to be taken up and has a greater influence on vaccination rates than general infection protection instruction. A certain biasing influence due to the different reference periods (vaccination counselling within 5 years vs. infection prevention instruction within 2 years) cannot be excluded at this point.

However, the prevalence of HBV infections in persons over 20 years of age has not been significantly reduced even after the introduction of vaccination (Kwon und Lee 2011). In addition various studies have shown that the response rate to HBV vaccination may depend on a number of factors^17–21^. Therefore, the immune status of persons at increased risk of infection should be assessed and, if necessary, boostered^22^.

In principle, the results clearly show the need for counselling and information. In their systematic review, Jarrett et al.^23^ state that the most effective way to increase vaccination rates is to target under-vaccinated members of specific populations with strategies that focus on increasing vaccination knowledge and awareness and to simplify access to vaccination.

### Limitations of this study

The application of a cross-sectional pattern basically does not allow reliable statements about cause-effect relationships. The study also fails to provide information on the number of vaccination doses administered (complete vs. incomplete basic immunisation vs. booster vaccination) or on the time at which HAV or HBV vaccination may have been given (e.g. being vaccinated as a child, before taking up employment or before starting career). The questionnaire did not contain validated questions, for it has been developed by the Institute of Teachers' Health. It was previously used in the special school study by Claus et al. (2014). Due to the retrospective data collection, bias in the information on HAV or HBV infection or vaccination cannot be excluded (recall bias). As a matter of principle, when interpreting the data and comparing them with other data sources, the relevant collection method must be taken into account (self-disclosure vs. vaccination record vs. determination of serostatus). The comparison of study participants and non-participants showed significant age differences. Corresponding age effects must therefore be taken into account when interpreting the data. However, the strength of this study lies in the high and representative participation rate.

## Conclusions

While nursing activities at special schools with support focus on physical and mental disabilities can certainly be described as belonging to the occupation, they occur comparatively rarely at schools with support focus on social-emotional disabilities. However, here too, the risk of both HAV or HBV infection cannot be ruled out due to occasional nursing activities, first aid activities and reported aggressive behaviour by pupils. Despite the widespread introduction of basic HBV immunization for infants and young children, this vaccination strategy alone does not appear to be sufficient to immunize the personnel concerned. Regular occupational health check-ups with inspection of the vaccination certificate or, if necessary, serological clarification of immunity and detailed vaccination counseling could contribute to an improvement in prevention through education, counseling and vaccination. In addition, at least the STIKO in Germany, as well as further European institutions, recommends that persons with a particularly high individual risk of exposure should be tested for anti-HBs after 10 years, followed by a booster vaccination if anti-HBs is lower 100 IU/l. Due to the increased risk of infection, occupational health care, including instruction on infection prevention and vaccination counselling concerning HAV and HBV, should be offered to all employees at all types of schools for handicapped pupils.

## Data Availability

The datasets used and/or analysed during the current study are available from the corresponding author.
